# The growing interests in Epstein–Barr virus: A bibliometric analysis of research trends, collaborations, and emerging hotspots

**DOI:** 10.1016/j.imj.2025.100194

**Published:** 2025-06-22

**Authors:** Lu Li, Jialin Wu, Jianghui Cai, Muhammad Arif Asghar, Rui Xiao, Jingwei Wu, Qinjian Zhao, Xiao Zhang

**Affiliations:** a College of Pharmacy, Chongqing Medical University, Chongqing 400016, China; b School of Medicine, University of Electronic Science and Technology of China, Chengdu 611731, Sichuan Province, China; c Department of Pharmacy, Chengdu Women's and Children's Central Hospital, School of Medicine, University of Electronic Science and Technology of China, Chengdu 611731, Sichuan Province, China; d Department of Epidemiology and Biostatistics, College of Public Health, Temple University, Philadelphia, PA 19122, United States

**Keywords:** Epstein–Barr virus, Bibliometrics analysis, Immunotherapy, Vaccine development, Pathogenesis

## Abstract

**Background:**

Epstein–Barr Virus (EBV) is a widespread human γ-herpesvirus linked to cancers and autoimmune diseases, but limited comprehensive bibliometric analysis appear to have been conducted in this field.

**Methods:**

Using Web of Science data, 16,318 EBV-related documents (2014–2023) were analyzed via VOSviewer, Bibliometrix, and Citespace following the Strengthening the Reporting of Observational Studies in Epidemiology reporting guideline.

**Results:**

This cross-sectional bibliometric analysis of 16,318 EBV-related documents (2014–2023) revealed a consistent upward trend in annual publications, reflecting growing global interest in EBV research. Collaborative networks demonstrated strong international partnerships, particularly between the United States and China. Keywords co-occurrence and burst analysis highlighted enduring focus on EBV pathogenesis, immune evasion mechanisms, and EBV-associated diseases like nasopharyngeal carcinoma, Hodgkin lymphoma, and multiple sclerosis. On the basis of this comprehensive bibliometric analysis, it showed that the emerging hotspots included immunotherapy, biomarkers, viral reactivation, and vaccine development, with clinical trials evaluating immune-checkpoint inhibitors of toripalimab, mRNA-based therapeutic vaccines targeting LMP2 and EBNA1, and prophylactic strategies such as glycoproteins-based ferritin nanoparticles or mRNA vaccines, indicating a shift toward precision interventions.

**Conclusions:**

EBV research has grown exponentially, driven by insights into structural-function relationships and immune evasion. Advances enable targeted prophylactic/therapeutic strategies. The analysis highlights needs to decode virus-host interactions, optimize vaccines, and translate findings clinically, aiming to raise disease awareness, guide immunotherapies, and reduce global health burdens.

## Highlights

• This bibliometric analysis reveals a notable rise in global attention toward EBV research, showcasing expanding scholarly engagement worldwide.
• The study outcomes systematically chart the intellectual terrain of EBV research, mapping key areas of knowledge and academic focus.
• This work delivers novel perspectives that can inform and inspire future investigations within the field of EBV, fostering innovative research directions.

## 1 Introduction

Epstein–Barr virus (EBV), also known as human herpesvirus 4 (HHV-4), is the first discovered human oncogenic virus and belongs to the γ-herpesvirus subfamily. It contains a double-stranded DNA genome of approximately 172 kb.^1^ The target cells of EBV infection include B cells, epithelial cells, natural killer/T (NK/T) cells, and macrophages.^2^ Because of the high infectivity and the common transmission routes mainly through saliva, more than 95% of adults are EBV carriers worldwide.^3^ Hence, EBV is an increasingly hot topic in current virology research.

Primary infection is typically asymptomatic in childhood, and nearly 35%–50% of the human adolescent population develop infectious mononucleosis approximately one month after infection, and the virus will persist throughout an individual's life.^4^ It was reported that EBV was originally found in Burkitt lymphoma in 1964,^5^ and later found to be associated with other types of lymphoma, such as Hodgkin lymphoma and NK/T cell lymphoma.^6^^,^^7^ Besides, EBV is also related to epithelial cancers, including nasopharyngeal carcinoma (NPC) and gastric carcinoma (GC).^8^ Therefore, the World Health Organization identified EBV as a class I oncogenic virus in 1997.^9^ The virus has evolved numerous epigenetic mechanisms by which it can affect its host and contribute to the development and progression of cancer.^10^ Regional prevalence of EBV-associated cancers is closely linked to specific malignancies, with NPC and GC highly prevalent in East Asia, Burkitt lymphoma in Africa, and Hodgkin lymphoma in developed countries.^8^ It is reported that the notable number of deaths, from nasopharyngeal cancer and Hodgkin lymphoma, was mainly attributed to EBV infections in the United States.^11^ Moreover, EBV is the pathogen of oral hairy leoplakia, systemic lupus erythematosus (SLE), rheumatoid arthritis (RA), and especially for multiple sclerosis (MS).^2^^,^^12^^,^^13^ Meanwhile, fatal mononucleosis, aplastic anemia and other serious diseases often occur after EBV infection in immunodeficient children. Overall, EBV infection has caused a severe disease burden. In the past 60 years, scientists have been committed to the research and development of vaccines and therapeutics against EBV infection and EBV-associated diseases. However, to date, no specific antiviral drugs or vaccines have been approved for clinical use.

Over the past decades, scientific advancements have significantly expanded our understanding of EBV and its associated diseases. However, these articles are too diverse to make us clearly define the focus of EBV-related researches and future directions. Besides, there is lack of enough useful information to track emerging scholars and hotspots in this field. Hence, given the burgeoning interests in this field and its potential benefits for public health, there is an urgent need for a comprehensive and systematic analysis of the current global research status about EBV. Unlike common systematic reviews, bibliometric analysis is a quantitative research analysis of scientific literatures to understand the development process and trends in this research field.^14^ It has become one of the most extensively employed methods for measuring and evaluating the quality, credibility, and impact of academic work.^15^ Therefore, we performed a bibliometric analysis to provide an overall knowledge map and emerging research areas which could enable the development of effective immunotherapies. Several emerging areas are driving the development of immunotherapies, including targeted treatments for EBV-associated cancers and autoimmune diseases. They include the better understanding of B- and T-cell epitopes, identification of potential vaccine targets to stimulate immune responses against EBV-associated malignancies, and development of monoclonal antibodies that neutralize key EBV proteins involved in cell entry and oncogenesis. These emerging areas hold significant potential for improving immunotherapy strategies targeting EBV-driven diseases, including cancers and immune-related disorders.

## 2 Materials and methods

The institutional review board of the Chongqing Medical University waived ethical approval, considering this was a bibliometric study. The data source used in this bibliometric study is publicly available. No protected health information was included. This cross-sectional study followed the Strengthening the Reporting of Observational Studies in Epidemiology (STROBE) reporting guideline^16^ and the reporting method described by Donthu et al.^17^ to combine the quantitative and qualitative assessments. The primary objective was to provide researchers and clinicians with a systematic knowledge mapping of the Epstein–Barr virus from 2014 to 2023.

### 2.1 Data source, search strategy and data retrieving

We conducted a systematic literature search of the Web of Science Core Collection (WoSCC) from January 1st, 2014, through December 31st, 2023. The WoSCC, widely acknowledged for its extensive coverage in scientometric analyses, enables plain text file export suitable for bibliometric studies.^18^ Two reviewers (Lu Li and Jianghui Cai) independently performed the literature search, if there are disagreements and ambiguities between them, they will be resolved through discussion or cross-checking consultation with another researcher (Rui Xiao). Combinations of the following Medical Subject Headings (MeSH) terms and keywords were used: “Epstein–Barr Virus”, “Epstein Barr Virus”, “Epstein–Barr Virus Infections”, “Epstein Barr Virus Infections”, “Epstein–Barr Virus Infection”, “Epstein Barr Virus Infection”, “E–B Virus”, “E B Virus”, “E–B Viruses”, “EBV Infections”, “EBV Infection”, “Virus Infection, Epstein–Barr”, “Virus Infections, Epstein–Barr”, “Infection, Epstein–Barr Virus”, “Infections, Epstein–Barr Virus”, “EBV”, “Herpesvirus 4, Human”, “Herpesvirus 4 (gamma), Human”, “HHV-4”, “Human Herpesvirus 4”, “Human Herpes Virus 4 Infections”, “Human Herpesvirus 4 Infections”, “Herpesvirus 4 Infections, Human”. The publication types were limited to articles or reviews, and no language restrictions was imposed. Duplicates were eliminated with CiteSpace (version 6.2.4). Scientific output data containing full records and cited references were exported from the WoSCC for the final analysis. The following bibliographic data was collected from eligible publications, including titles, abstracts, first authors, corresponding authors, affiliations, countries/regions, publication years, keywords, number of citations, journals, impact factor (IF), and Journal citation reports (JCR) division of journals.

### 2.2 Eligible criteria

The inclusion criteria for our study were as follows: (1) articles involving Epstein–Barr virus, EBV, Epstein–Barr Virus Infections, or herpesvirus 4, human; (2) article type: original article, review, or research letter (including research data); (3) article language: English.

Exclusion criteria were as follows: (1) article type: letter/correspondence (without research data), editorial, opinion, comment, meeting/conference abstract, correction/erratum, retraction; (2) the documents marked with Early Access were also excluded because of the missing of the specific publication year and date information.

### 2.3 Bibliometric analysis

The VOSviewer version 1.6.19 (https://www.vosviewer.com/), Citespace version 6.2.4 (https://citespace.podia.com/), and Bibliometrix package version 4.3.1 (https://bibliometric.com/) software were applied to conduct the bibliometrics and visualization analysis.

The VOSviewer software was used to construct and visualize the bibliometric networks of co-authorship, co-occurrence, co-citation, and bibliographic-coupling.^19^ In the analysis, the co-authorship networks were used to explore the collaborative relationships between authors and institutions.^20^ The co-occurrence network shows how the keywords are related. The co-citation and bibliographic-coupling were constructed to identify research clusters and intellectual structures in disciplines.^21^^,^^22^ Additionally, an international collaboration between countries was visualized.

The Citespace package was used to construct and visualize the burst terms, citation bursts, and dual-map overlay.^23^ The time span is “2014–2023” in a 1-year time duration. This study presented both burst terms and citation bursts to identify keywords that experienced high citation bursts during a particular period. The dual-map overlay was constructed to evaluate the patterns of connections and movements within multiple disciplines.^24^ The Bibliometrix package was used to determine the frequency of trend topics.^25^

## 3 Results

### 3.1 The summarization of included studies and publications

**Fig. 1:**
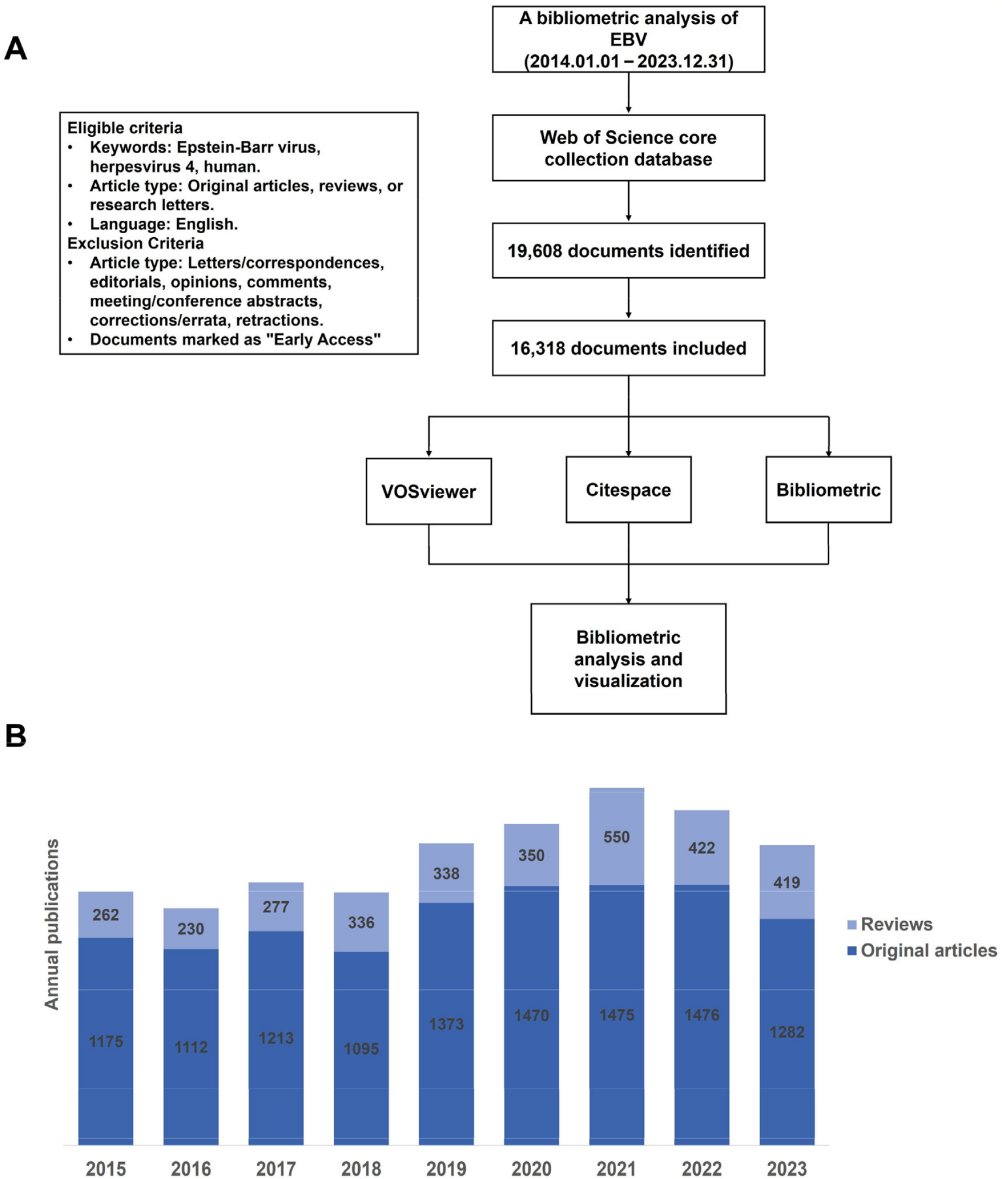
(A) Flow chart of study selection and bibliometric analysis. (B) Trend of annual publications over time.

A total of 19,608 records on EBV research were retrieved in the WoSCC from January 1st, 2014, to December 31, 2023. According to the eligible criteria, we finally included 16,318 documents in our study, among which 12,884 (78.96%) were original articles and 3,434 (21.04%) were reviews. The flow chart for this bibliometric analysis is shown in Fig. 1A. The highest numbers of published articles were in the field of Oncology (*n* = 3,062, 18.76%), followed by Immunology (*n* = 1,985, 12.16%) and Virology (*n* = 1,468, 9.00%). The subject categories of EBV research are listed in the Supplementary files, Fig. S1. In addition, Fig. 1B shows the annual number of published papers on EBV research. In the past 10 years, great attention has been paid to EBV research. The annual number of publications is gradually increasing, particularly during 2016–2021, from 1,342 in 2016 to 2,025 in 2021.

### 3.2 Co-authorship analysis

**Fig. 2:**
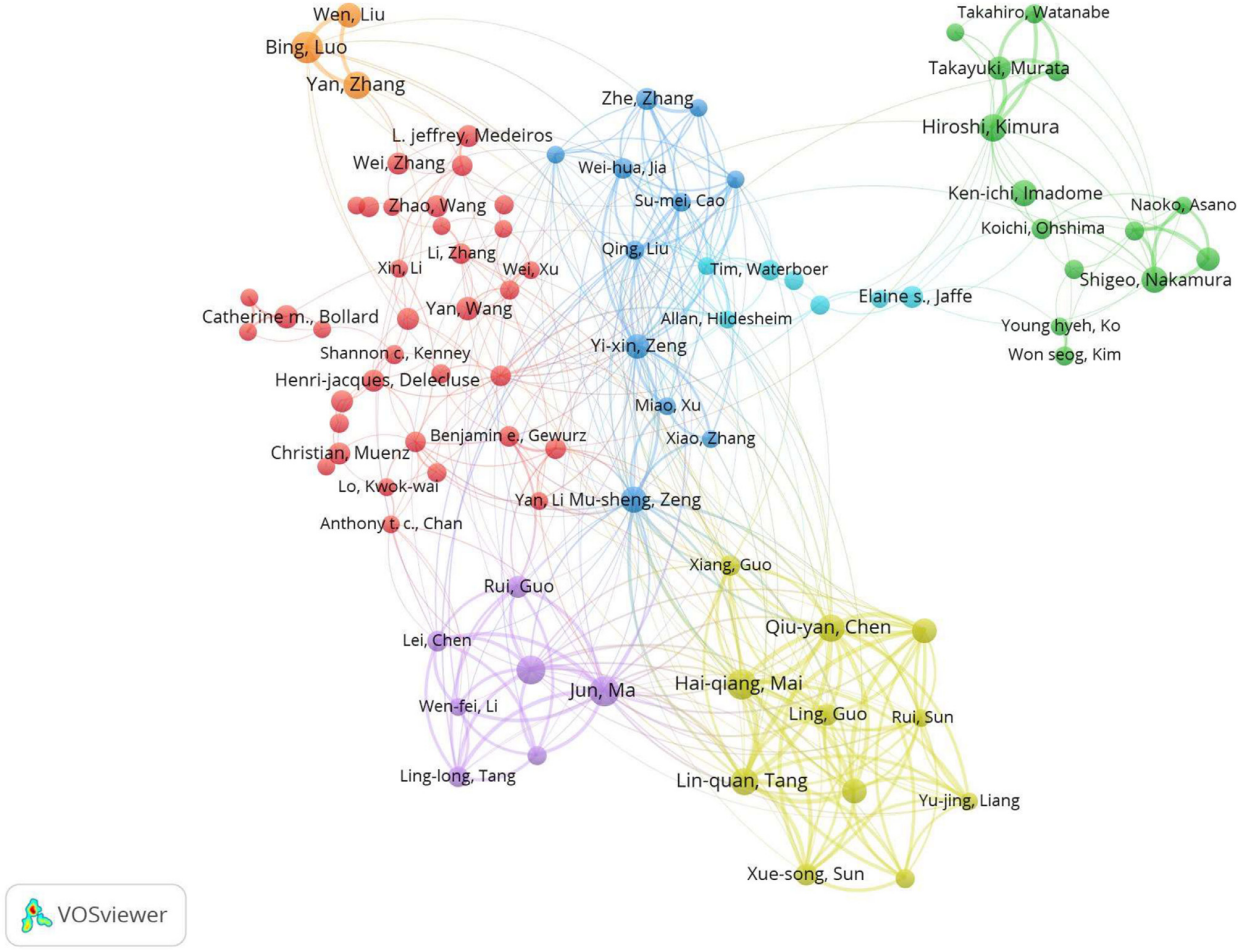
Network map of co-authorship among authors who published more than 25 articles. Different colors represent different clusters, and the cluster represents a collaborative network of authors who have published more than 25 articles. The thickness of lines indicates the strength of the relationship between authors relative to others.

A co-authorship analysis was performed on all publications to depict the collaborations between authors who have jointly contributed to these scholarly publications. In the network visualization, a total of 94 authors who published more than 25 papers formed a collaborative network and were divided into 7 clusters (Fig. 2). The thickness of lines indicates the strength of the relationship between authors relative to others. The largest cluster (Cluster 1, in red) contains 35 items, and the smallest cluster includes 3 items. Cluster 2 (in green) is primarily comprised of researchers from Qingdao University, with its core figure being Prof. Bing Luo. Cluster 2 also has the largest nodes (Bing Luo) and is composed of the most active coauthors in the field. The top three authors who published the highest number of studies during the last 10 years were Bing Luo, Hai-qiang Mai, and Jun Ma, with 80, 72, and 72 articles, respectively (Table 1). Half of the top 10 productive authors are from Sun Yat-sen University. The overlay visualization of co-authorship shows that some authors have a quick growth in the number of papers published recently, such as Xue-song Sun. More specific information can be obtained from the Supplementary files, Fig. S2.

**Table 1:** Leading contributors to EBV research based on publication volume (2014–2023).

Rank	Author	Affiliation	Documents	Citations
1	Bing Luo	Qingdao University	80	460
2	Hai-qiang Mai	Sun Yat-sen University	72	1271
3	Jun Ma	Central South University	72	3422
4	Ying Sun	Sun Yat-sen University	66	3386
5	Hiroshi Kimura	Nagoya University	63	1149
6	Lin-quan Tang	Sun Yat-sen University	62	966
7	Qiu-yan Chen	Sun Yat-sen University	66	1162
8	Mu-sheng Zeng	Sun Yat-sen University	57	1564
9	Shigeo Nakamura	Nagoya University Hospital	55	814
10	Ken-ichi Imadome	National Center for Child Health and Development	54	433

### 3.3 Contributions of countries

A total of 145 countries/regions and 11,501 organizations participated in the EBV research in the past 10 years. Seventy-five countries/regions that have published at least 15 documents formed the 6 clusters in the network map of co-authorship (Supplementary files, Fig. S3A). The network map also revealed the tight communications between these countries/regions. The largest cluster (in red) had 27 items, and the smallest cluster had 3 items. The United States was the most productive country based on the number of publications (*n* = 5,263), followed by China (*n* = 4,178) and Japan (*n* = 1,445), respectively. The combined number of publications from the top 3 productive countries accounted for more than half of the documents related to EBV research.

**Fig. 3:**
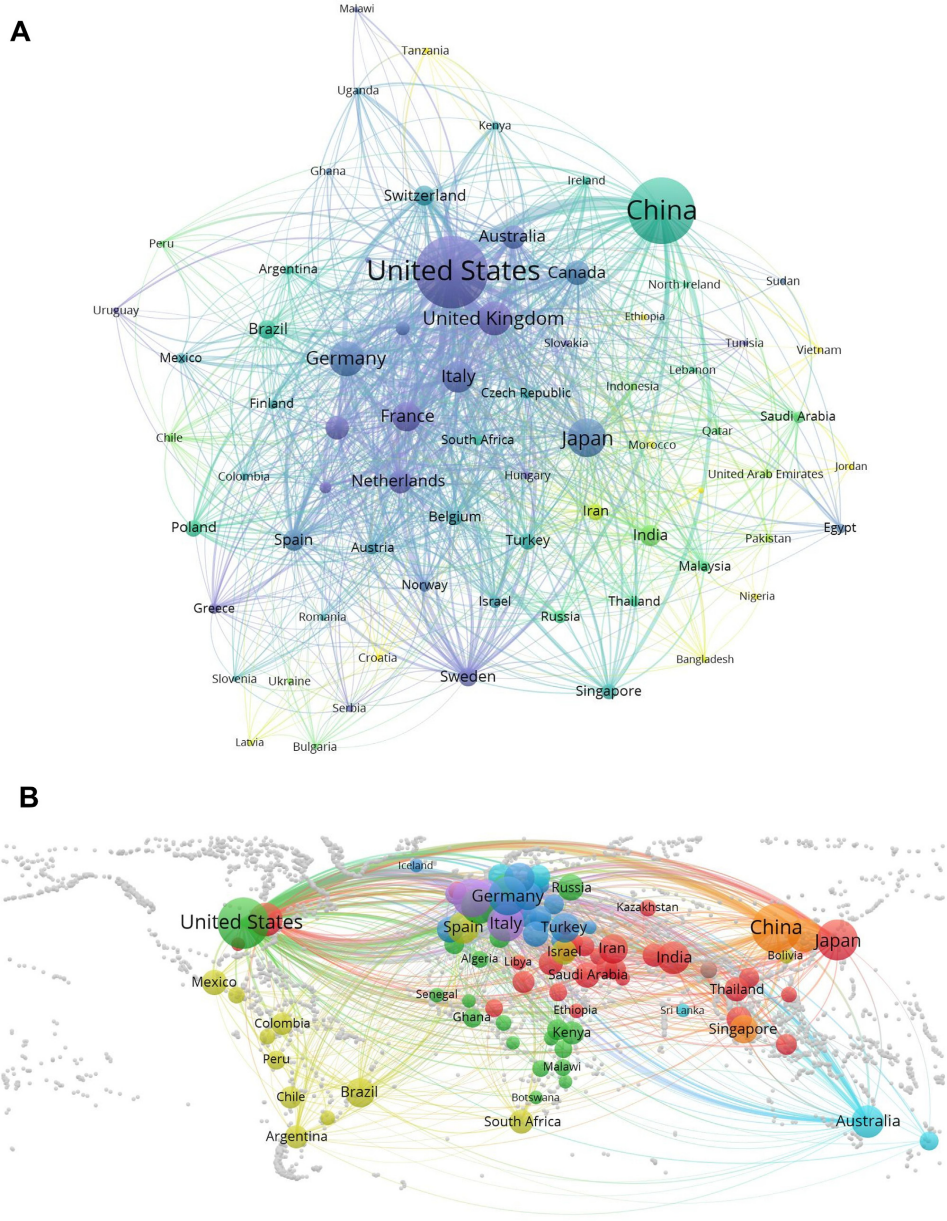
(A) Item density visualization of co-authorship among countries. (B) The global collaboration map.

Furthermore, the three outstanding countries whose publications on EBV received the highest number of citations were the United States (Total Citation, TC = 144,430), China (TC = 70,471), and the United Kingdom (TC = 34,998), respectively. Table 2 summarizes the top 10 countries/regions for publications and citations related to EBV. Some countries, such as China, have shown an increasing number of publications in recent years (Fig. 3A). Notably, there are lots of active cooperations between different countries, and the United States plays a strong bridge role among these cooperations (Fig. 3B). Intense collaborations between countries/regions resulted in thicker connecting lines between countries. The United States has maintained a robust national collaboration with China (Supplementary files, Fig. S3B). In addition, Germany, the United Kingdom, and Japan are also important nodes of collaboration.

**Table 2:** Top 10 countries/regions for publications and citations related to EBV.

Rank	Countries/regions	Documents, *n* (%)	Citations	Average citations
1	United States	5263 (32.25%)	144,430	27.44
2	China	4178 (25.60%)	70,471	16.87
3	Japan	1445 (8.86%)	23,112	16.00
4	Germany	1222 (7.49%)	29,577	24.20
5	United Kingdom	1129 (6.92%)	34,998	31.00
6	Italy	1002 (6.14%)	24,295	24.25
7	France	837 (5.13%)	23,418	27.98
8	Canada	591 (3.62%)	19,440	32.89
9	Republic of Korea	567 (3.47%)	16,537	29.17
10	Australia	550 (3.37%)	18,558	33.74

### 3.4 Contributions of Institutions

To further discover the publication patterns among different institutions, we presented the top ten institutions' results according to their total number of publications in Table 3. Sun Yat-sen University (*n* = 714, 4.04%) and National Cancer Institute (*n* = 245, 1.38%) had the highest number of publications during the study period, followed by the University of Hong Kong (*n* = 225, 1.27%), Harvard Medical School (*n* = 210, 1.18%), and Karolinska Institutet (*n* = 207, 1.17%). The 10 most productive EBV research institutions in the world were in the USA, China, and Sweden. This institution analysis reveals that the 10 most productive organizations conduct more in-depth research and play an essential role in the field of EBV.

**Table 3:** The top 10 institutions in term of the publications in EBV research.

Rank	Institutions	Documents, *n* (%)	Citations	Average citations
1	Sun Yat-sen University	714 (4.38%)	16,518	23.04
2	National Cancer Institute	245 (1.50%)	9,321	38.05
3	The University of Hong Kong	225 (1.38%)	7,694	34.20
4	Harvard Medical School	210 (1.29%)	7,327	34.89
5	Karolinska Institutet	207 (1.27%)	5,603	27.07
6	Stanford University	206 (1.26%)	8,702	42.24
7	University of Pennsylvania	180 (1.10%)	5,272	29.29
8	University of Texas MD Anderson Cancer Center	179 (1.10%)	6,417	35.85
9	Fudan University	166 (1.02%)	2,687	16.19
10	The Chinese University of Hong Kong	163 (1.00%)	9,484	58.18

### 3.5 Journal distribution analysis and bibliographic coupling

**Fig. 4:**
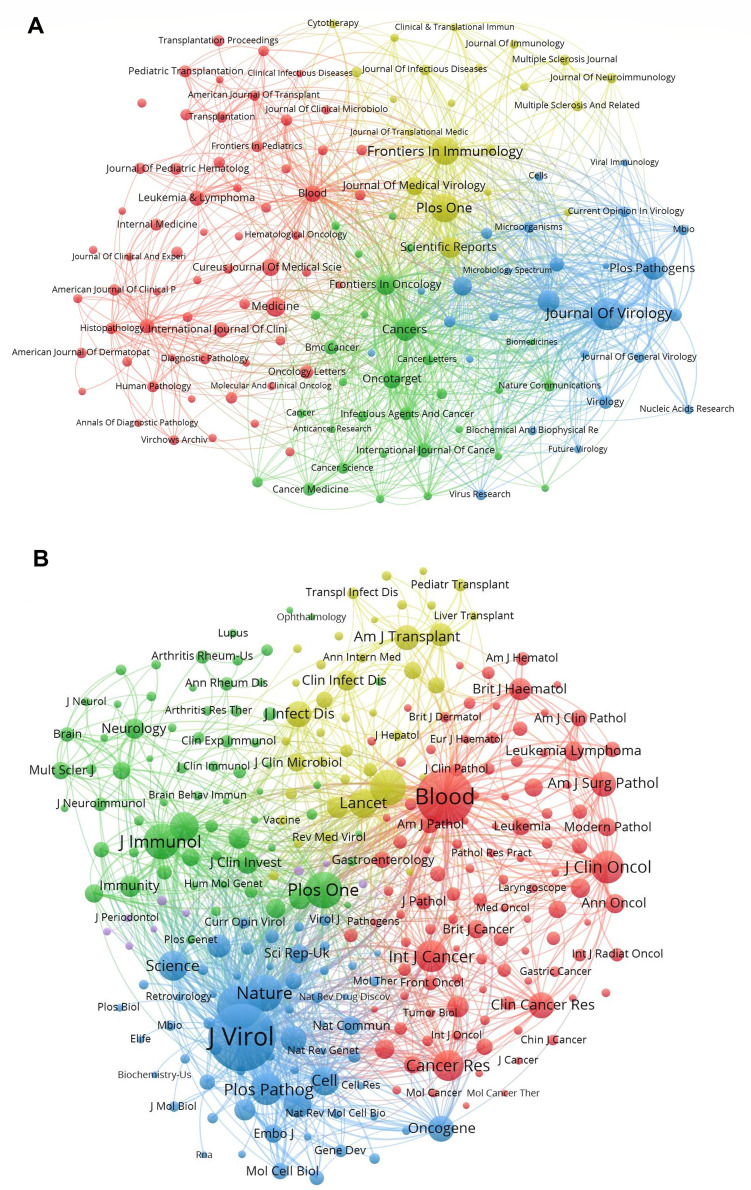
(A) Network map of citation among journals. (B) Network visualization of co-citation among journals.

The journal analysis revealed that 2,844 journals were involved in EBV research during the study period (Fig. 4A). The top 10 journals are shown in Supplementary files, Table S1, *Journal of Virology* was the most influential journal with the highest number of publications and total citations (*n* = 398, TC = 10,051), followed by *Frontiers in Immunology* (*n* = 360, TC = 7,060), *Plos One* (*n* = 360, TC = 8,484), *Cancers* (*n* = 219, TC = 2,272), and *Plos Pathogens* (*n* = 206, TC = 6,488). It is worth noting that *Blood* published 108 articles and received the third most total citations (TC = 7,991). Four of the top 10 journals were classified in JCR Quartile 1 (Q1), five were Q2, and one was Q3. The publishers of the top 10 journals were from the United States, United Kingdom, and Switzerland. The bibliographic coupling map of sources is shown in the Supplementary files, Fig. S4A, which visually highlights the relationships between scholarly sources based on their co-citation patterns. The top three journals with the highest total link intensity were: *Journal of Virology* (246,732 times), *Cancers* (207,323 times), and *Frontiers in Immunology* (173,583 times). These journals connected the majority of the articles in EBV.

The cited journals network provided insights into which journals are frequently cited together, indicating thematic or subject-related connections between them. Each size of the node in the network visualization represents the number of co-citations. As shown in Fig. 4B, journals with a minimum co-citation equal to 500 were filtered to map, composed of 5 clusters in the co-citation network. It is clear to see that *Journal of Virology* has positive co-citation relationships with *Virology* and *Nature*, among others. The dual-map overlay of journals found that the four main citation paths were Molecular/Biology/Immunology, Medicine/Medical/Clinical to Molecular/Biology/Genetics, and Health/Nursing/Medicine (Supplementary files, Fig. S4B).

### 3.6 Co-occurrence of keywords, burst keywords, and trend topics

**Fig. 5:**
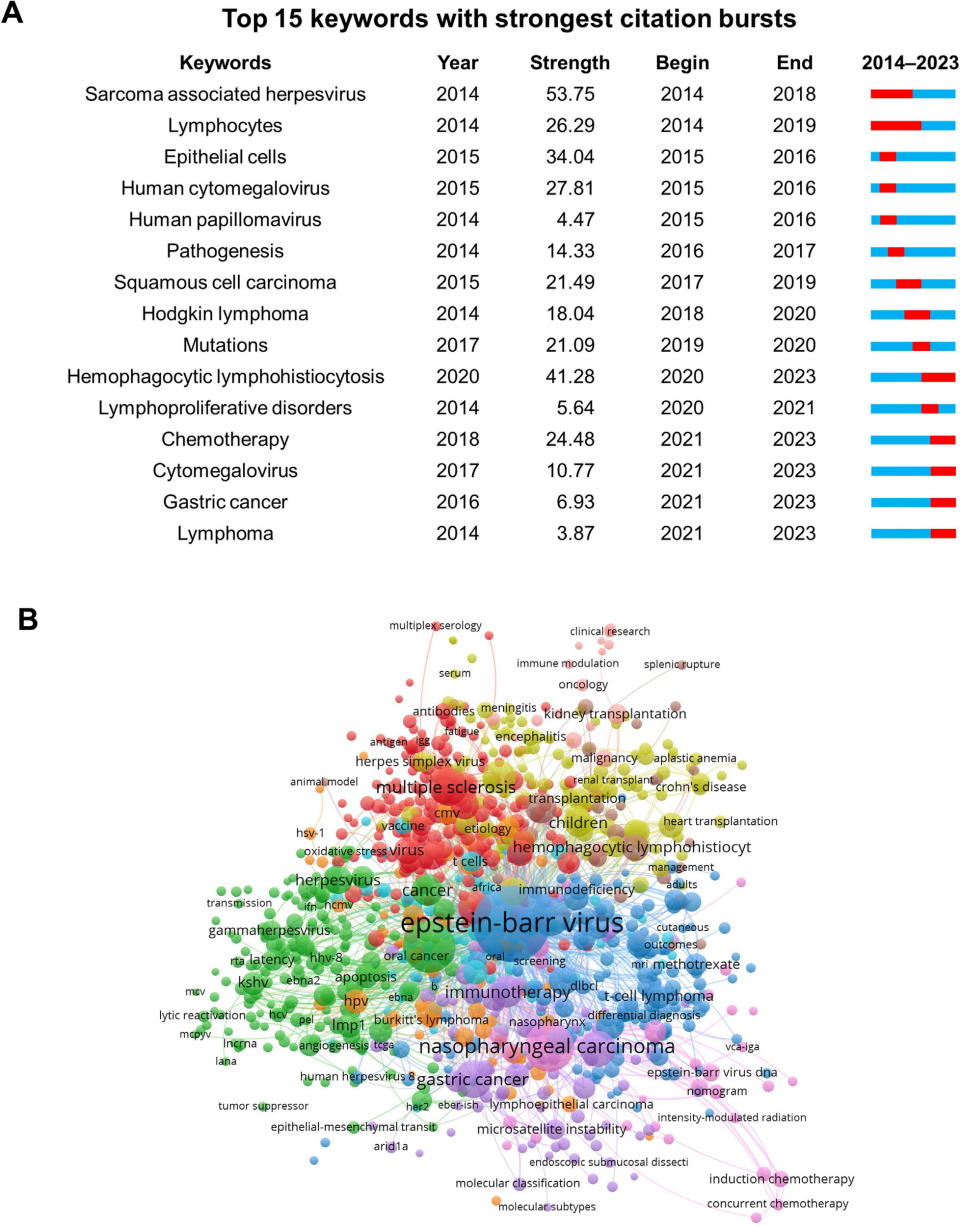
(A) Bursts citation analysis of keywords. (B) Co-occurrence of keywords. Different colors are used to distinguish different clusters, representing a set of keywords closely related in theme or concept, forming different sub-research fields. Lines represent the co-occurrence relationships between keywords, and their thickness or color shade reflects the co-occurrence strength. The thicker or darker the line, the higher the co-occurrence frequency and the closer the association between keywords, intuitively presenting the tightness of co-occurrence of keywords in the literature and the division of thematic clusters.

In Fig. 5A, we present the top 15 keywords with the most robust citation bursts, with a minimum duration of one year. The keyword “lymphocyte” (2014–2019) has received the most protracted attention over time. Meanwhile, keywords such as “chemotherapy” (2021–2023), “cytomegalovirus” (2020–2023), and “gastric cancer” (2020–2023) have been used more recently, indicating that these keywords have attracted enough attention to become popular research topics in the future. The trend topic map (Supplementary files, Fig. S5) revealed that some keywords had maintained high frequencies in recent years and might predict the future trend of the EBV study. Some of them focused on the pathogenesis and therapeutic mechanisms of EBV (e.g., “apoptosis”, “rituximab”, “PD-L1”, “immunotherapy”), and some focused on EBV-associated disease (e.g., “multiple sclerosis”, “gastric cancer”). Additional focus has been put on the clinical management of EBV or related diseases (e.g., “prognosis”). It is worth noting that the trending topic shifted to “SARS-CoV-2” and “COVID-19” after the onset of the COVID-19 pandemic in 2019.

The co-occurrence of keywords (Fig. 5B) revealed that Epstein–Barr virus (*n* = 9,531), expression (*n* = 1,911), and infection (*n* = 1,854) were the most co-occurrence of keywords. Both the clusters of keywords (Supplementary files, Fig. S6A) and the timeline map (Supplementary files, Fig. S6B) showed that these keywords mainly formed 5 clusters. The cluster #0 Epstein–Barr virus and the cluster #4 human papillomavirus were primarily associated with the risk factors, epidemiology, and prevalence of EBV/human papillomavirus. The cluster #1 gene expression and the cluster #3 expression involved the pathological processes and molecules of EBV, including B cells, NF-κB, expression and replication. The cluster #2 multiple sclerosis and cluster #6 Hodgkin lymphoma described the EBV-associated diseases. According to the results of analys, Table S2 represents current research hotspots and article contents in EBV research field.

## 4 Discussion

In this study, we performed a bibliometric analysis of the research papers on EBV in 2014–2023 from the WoSCC to comprehensively describe the global research status of EBV over the last decade and identify the research hotspots and future trends. Our results showed a gradually increasing number of EBV research in the past 10 years. The United States, China, and Japan were the most productive countries, and part of the discrepancy in the quantity of publications across different countries can be explained by the factors of economy or population. Sun Yat-sen University in China as well as the National Cancer Institute in the United States published most papers during the study period, both of them are well-done institutions in cancer research and have published many excellent high-quality articles on EBV-induced cancers. In terms of journals, *Journal of Virology* (IF: 4, Q2) was the most influential journal with the maximum number of publications and total citations, it is well known that highly cited literatures have received a lot of attention in the research field, which is well worth learning for beginners.

To our knowledge, relatively few studies have thus far undertaken a bibliometric analysis of highly cited research related to EBV. Keywords cooccurrence analysis can further reveal the research hotspots and development tendency in a scientific field. Through the keyword analysis, we supposed that the pathogenesis of EBV would attract increasing attention in the future.

It is well known that EBV can establish a lifelong latent period post primary infection and is adept at evading the host immune responses.^26^ When EBV remains latent in the host cells, it only expresses a small number of viral proteins, thereby avoiding activating the host immune response. Besides, many other mechanisms were also utilized by EBV to evade the host immune response. Firstly, EBV can induce host cells to express immunosuppressive molecules such as programmed death ligand-1 (PD-L1) to inhibit T cell function.^27^ Meanwhile, it can also interfere with the antigen presentation process and prevent viral peptides from binding to MHC molecules, thus avoiding T cells recognition.^27^ Moreover, EBV can be encapsulated in certain extracellular vesicles, such as exosomes, which may help the virus evade attack by the immune system.^28^^,^^29^ In addition, numerous mechanisms are worth studying, such as inducing cell apoptosis resistance, mutations in the viral genome, which are the prevalent focus of EBV research recently.^30^, ^31^, ^32^

On the other hand, “immunotherapy” also acts as the primary keyword of emerging research hotspots. Immunotherapy activates the immune response in the tumor microenvironment by altering the biological properties of immune effector cells, thereby inhibiting or killing cancer cells.^33^ Currently, the research hotspots of EBV immunotherapies include immune-checkpoint inhibitors (ICI) and cancer vaccines.^34^ With a deeper understanding of the carcinogenic mechanism and the infection process of EBV, the targets used for the development of intervention strategies are gradually clarified and confirmed. In addition, significant progresses have also been made in advanced immunotherapy technologies and rational vaccine design. For example, studies have shown that NPC patients are suitable for immunotherapy for the following reasons: expression of EBV antigen and CD4^+^/CD8^+^ T cell target proteins, abundant lymphocyte infiltration, high expression of PD-L1, and the presence of several crucial immune molecules (CD40, CD70, CD80, and CD86) that regulate T-cell activation.^35^, ^36^, ^37^, ^38^, ^39^, ^40^ Recently, ICIs targeting PD-1 gradually become a promising treatment approach to improve the prognosis of NPC patients. In 2021, compared to placebo combined with chemotherapy, Mai et al.^41^ found that the addition of the anti-PD-1 antibody toripalimab to chemotherapy has been shown to improve outcomes in patients with NPC, reducing the risk of progression or death by 59%. Besides, the development of vaccines, both prophylactic and therapeutic, is also receiving increasing attention from researchers (Fig. S7). The Epstein–Barr virus nuclear antigens (EBNAs) and latent membrane proteins (LMPs) are usually preferred vaccine targets for immunotherapeutic. They can induce specific cellular immune response, especially for the cytotoxic T cells to kill EBV-infected tumors.^42^^,^^43^ In the past two decades, a variety of EBV therapeutic vaccines have entered clinical trials to prevent disease^44^, ^45^, ^46^, ^47^ (Table S3). A modified vaccinia Ankara vector expressing the carboxyl terminus of EBNA1 and full-length LMP2 was developed to treat patients with NPC. This vaccine was evaluated in patients in the United Kingdom and in Hong Kong, China with NPC and induced specific CD4^+^ and CD8^+^ T cell responses to EBNA1 and LMP2, respectively.^45^^,^^46^ Another therapeutic EBV vaccine, an adenovirus vector expressing LMP2, was tested in a phase I trial of patients with NPC in China.^48^ More recently, new vaccine formulations were prepared and tested in clinical trials including DC- and mRNA-based therapeutic vaccines against EBV-associated diseases such as NPC or NK/T lymphoma (NCT02115126, NCT05831111 and NCT05714748). Based on our analysis, we speculate that and rational design of immunogen and the selection of effective adjuvants for potential vaccine development will be the focus of future research on EBV.

In addition, successful prophylactic vaccines against EBV infection will solve the public health burden caused by EBV infection. As reported, a mRNA-based vaccine termed mRNA-1189 developed by Moderna Inc is being evaluated in clinical trial phase I (NCT05164094). The other two on-going clinical trials are intended to test the efficacy of Ferritin-based nanoparticle vaccine (NCT04645147 and NCT05683834) in which recombinant gp350 was used as the vaccine immunogen (Table S4). Therefore, the immune intervention strategies including vaccines and therapeutics against EBV or associated diseases will become the most important hotspots in the following years.

Based on the comprehensive analysis, we researched recently published papers in EBV field. Table S2 showed the representative papers in the mentioned topics. Prophylactic vaccines are being developed especially based on the deep understanding on immunedominant epitopes of multiple glyproteins such as gB, gHgL and gp42. Screening and charactereutralizing of antibodies significantly promote the rational design of immunogens. Of course, research on the process of viral infection and its cofactors and receptors is indispensable for the verification of key targets for prophylactic vaccines. Interaction between EBV and host cells in NPC or GC patients will aid the finding of potential pathogensis mechanisms that may become ideal targets for drug design and intervention. In addition, with the determination of viral protein structures, structure-guided design of small molecule drug also become a hot topic especially targeting EBNA1 and LMP1. Finally, it is proven that EBV infection is tightly associated with various autoimmune diseases including MS, RA and SLE. The complicated pathogenesis mechanisms except molecular mimicry are being investigated.

The next five years in EBV research are set to revolutionize the landscape of immunotherapies, offering targeted vaccines (e.g., targeting latent EBV antigens such as EBNA1, LMP1, and LMP2 to stimulate immune responses against EBV-driven malignancies and strategies to disrupt EBV latency), novel T-cell-based therapies (including mRNA based vaccines), and strategies to disrupt EBV latency. These advancements will provide more effective, personalized treatments for EBV-associated diseases, ultimately improving patient outcomes and shaping the future of viral oncology and immunotherapy.

## 5 Conclusions

This bibliometric analysis highlights the increasing global focus on EBV research, particularly in the context of immunotherapy. Over the past decade, there has been a surge in studies exploring EBV's role in carcinogenesis, autoimmune diseases, and immune evasion mechanisms, leading to promising advances in immunotherapeutic strategies such as T-cell-based therapies, immune checkpoint inhibitors, monoclonal antibodies, and therapeutic vaccines. The growing international collaborations and interdisciplinary approaches are accelerating the development of novel prophylactic and therapeutic interventions. As EBV research continues to evolve, the integration of emerging technologies-including mRNA vaccines, CRISPR-based gene editing, and personalized medicine approaches-is expected to revolutionize EBV-targeted immunotherapies. With 60 years of progress since EBV discovery, the next five years hold immense potential for breakthroughs in immunotherapy, bringing us closer to effective treatments and vaccines against EBV-associated diseases.

## Informed consent

Not applicable.

## Organ donation

Not applicable.

## Ethical statement

The article does not involve patient data.

## Data availability

The data supporting the findings of this study are openly available in the WoS database.

## Animal treatment

Not applicable.

## Generative AI

Not applicable.

## Funding

The work was supported by Chongqing Municipal Scientific Research and Innovation Projects (CYS23359 and CYS240331).

## Declaration of competing interest

The authors declare that they have no known competing financial interests or personal relationships that could have appeared to influence the work reported in this paper.

## Acknowledgements

None.

## CRediT authorship contribution statement

**Lu Li:** Writing – original draft, Data curation, Conceptualization. **Jialin Wu:** Writing – original draft, Data curation, Conceptualization. **Jianghui Cai:** Writing – original draft, Visualization, Validation, Formal analysis. **Muhammad Arif Asghar:** Supervision, Software, Investigation. **Rui Xiao:** Writing – review & editing, Investigation. **Jingwei Wu:** Writing – review & editing, Investigation. **Qinjian Zhao:** Resources, Methodology. **Xiao Zhang:** Resources, Methodology, Funding acquisition.

## Appendix Supplementary materials

Image, application 1
