# Supplementary material for: The growing interests in Epstein–Barr virus: A bibliometric analysis of research trends, collaborations, and emerging hotspots
Source: Infect Med (Beijing). 2025 Jun 22;4(3):100194. doi: 10.1016/j.imj.2025.100194 (PMC12284522; doi:10.1016/j.imj.2025.100194)
Supplement: Supplementary file 1 [file mmc1.docx]

**Supplementary Figures:**

**Fig. S1.** Treemap chart of WoS categories.

**Fig. S2.** The overlay visualization of co-authorship.

**Fig. S3.** Network map (A) and chord diagram of co-authorship (B) among countries.

**Fig. S4.** Bibliographic-coupling of sources (A) and the dual-map overlay of journals related to EBV research (B).

**Fig. S5.** Trend topic of EBV.

**Fig. S6.** Clusters of keywords (A) and timeline of keywords (B).

**Fig. S7.** The number of research articles and clinical trials about EBV field (1964–2024).

**Supplementary Tables:**

**Table S1** The most 10 journals that published EBV researches.

**Table S2** Insights from recent studies.

**Table S3** Clinical trials for therapeutic vaccines targeting EBV.

**Table S4** Clinical trials for prophylactic vaccines against EBV.

**Fig. S1.** Treemap chart of WoS categories.


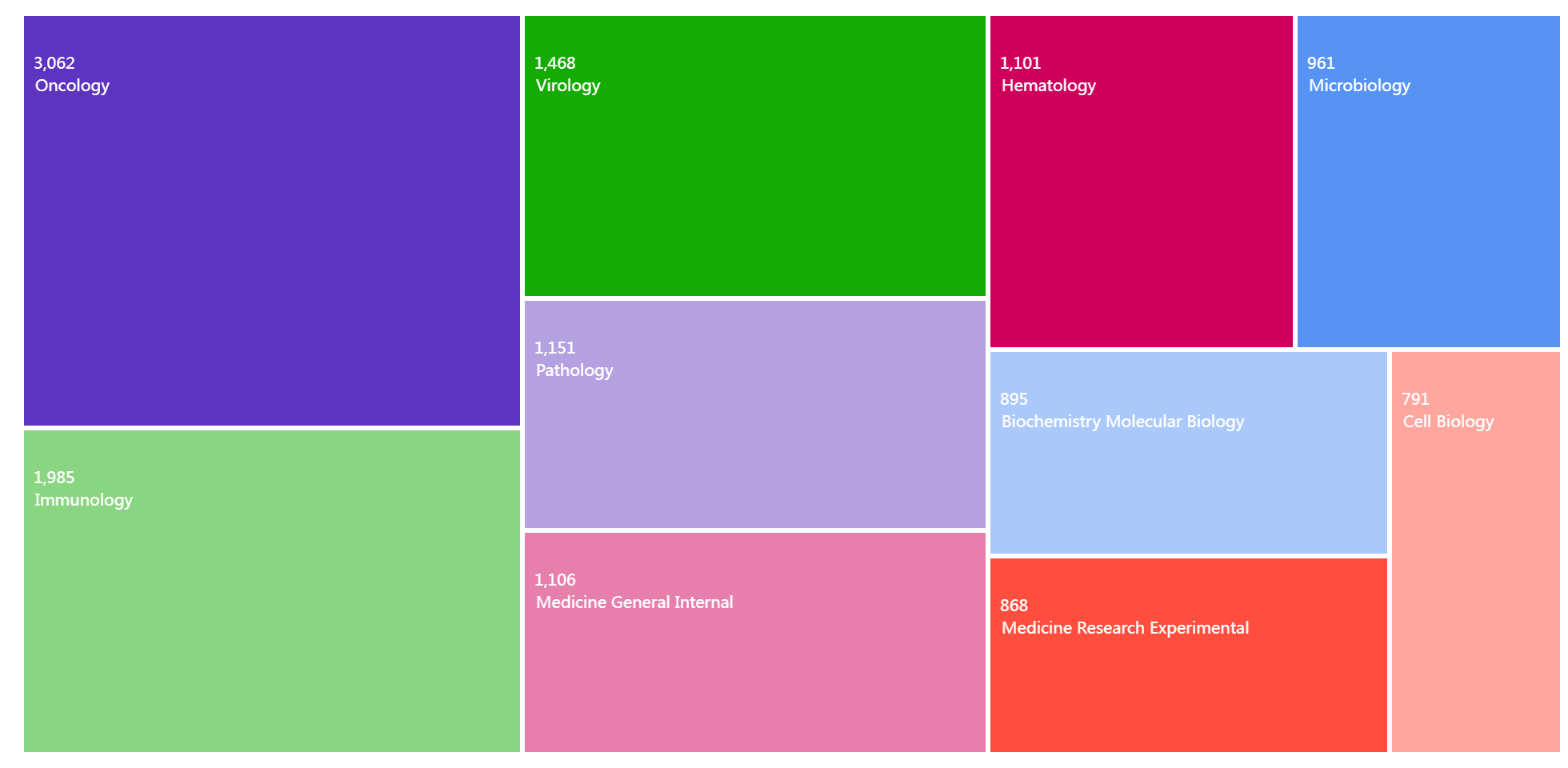


**Fig. S2.** The overlay visualization of co-authorship.

**
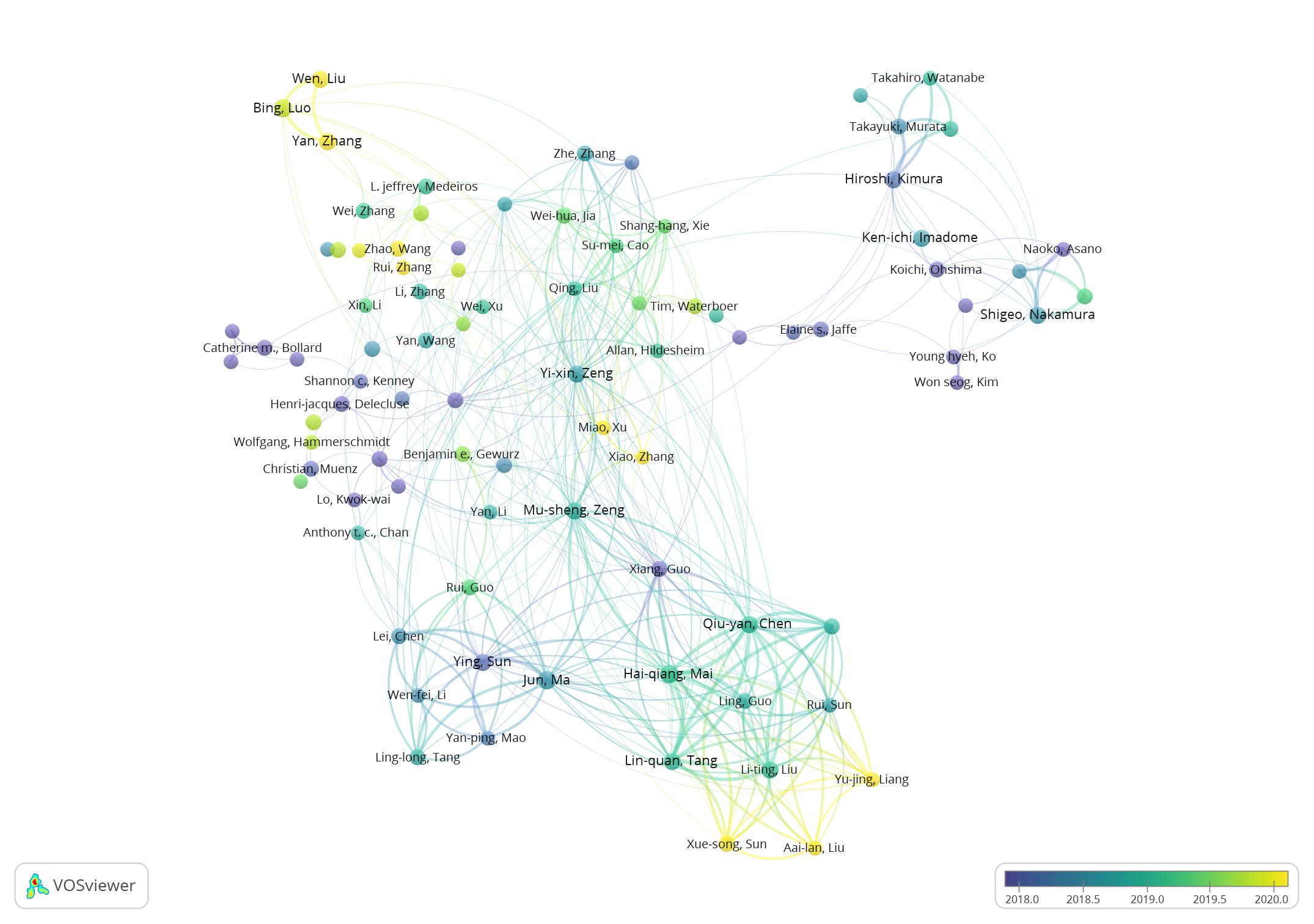
**

**Fig. S3.** Network map (A) and chord diagram of co-authorship (B) among countries.

**
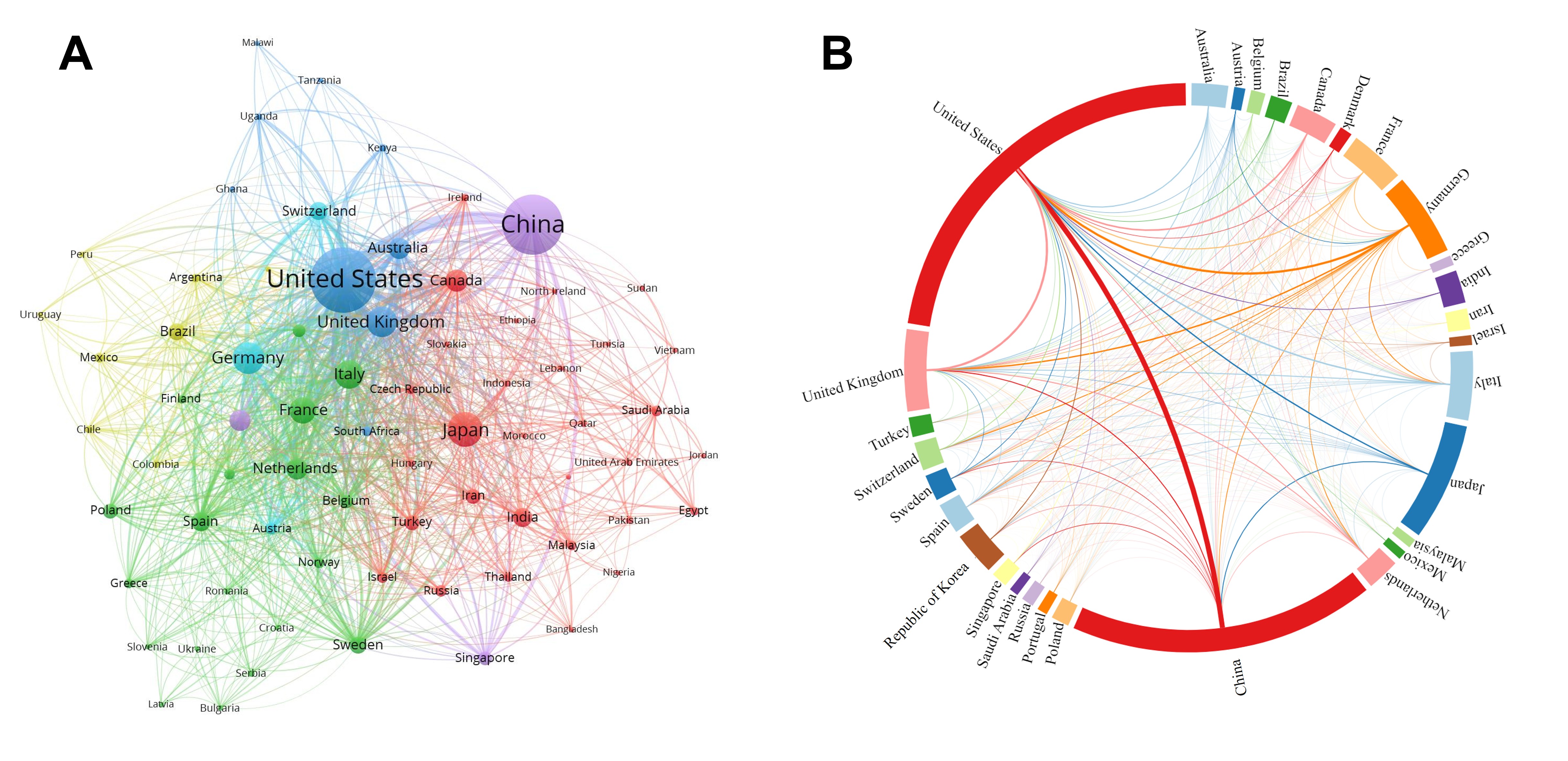
**

**Fig. S4.** Bibliographic-coupling of sources (A) and the dual-map overlay of journals related to EBV research (B).

**
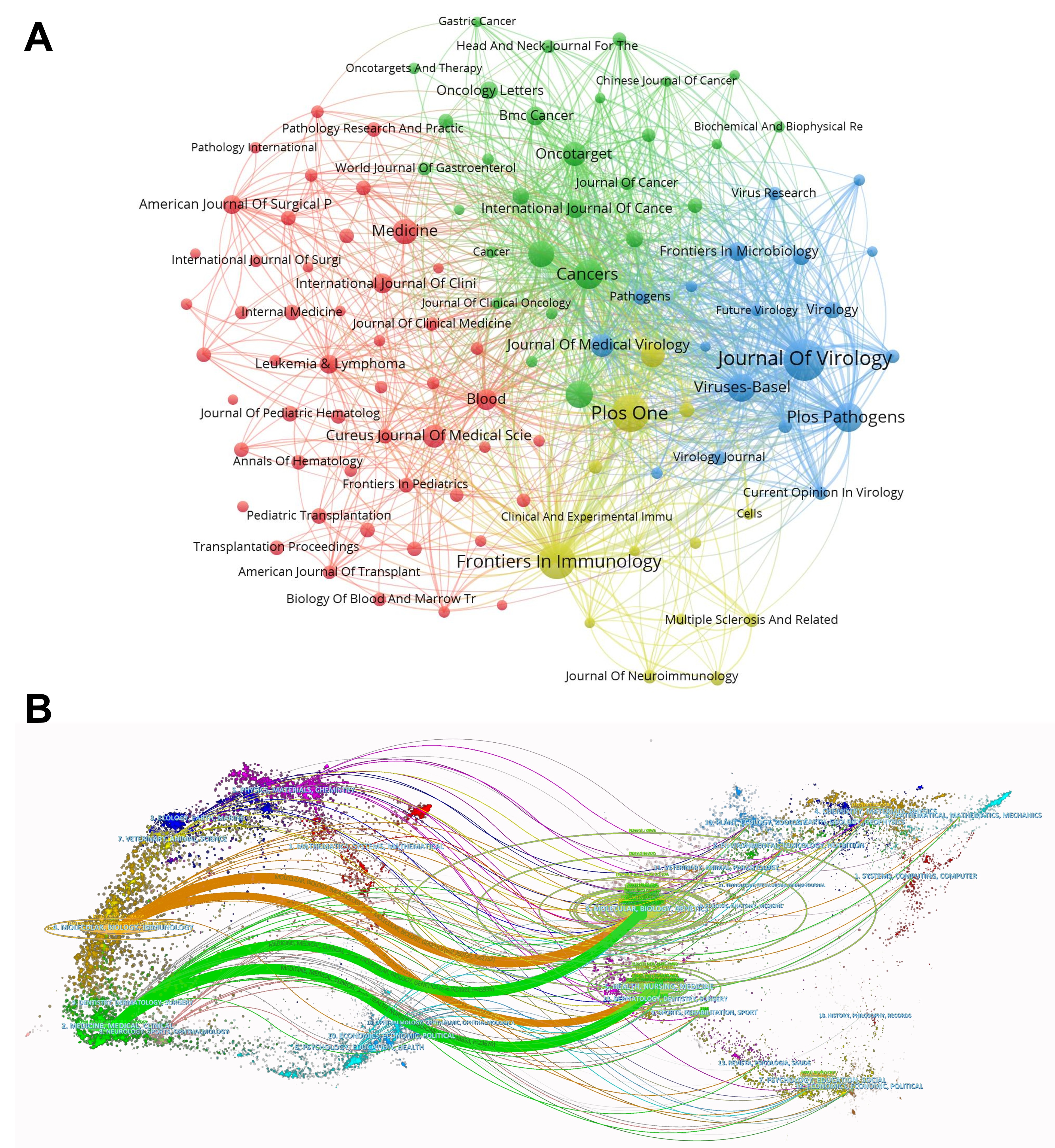
**

**Fig. S5.** Trend topic of EBV.

**
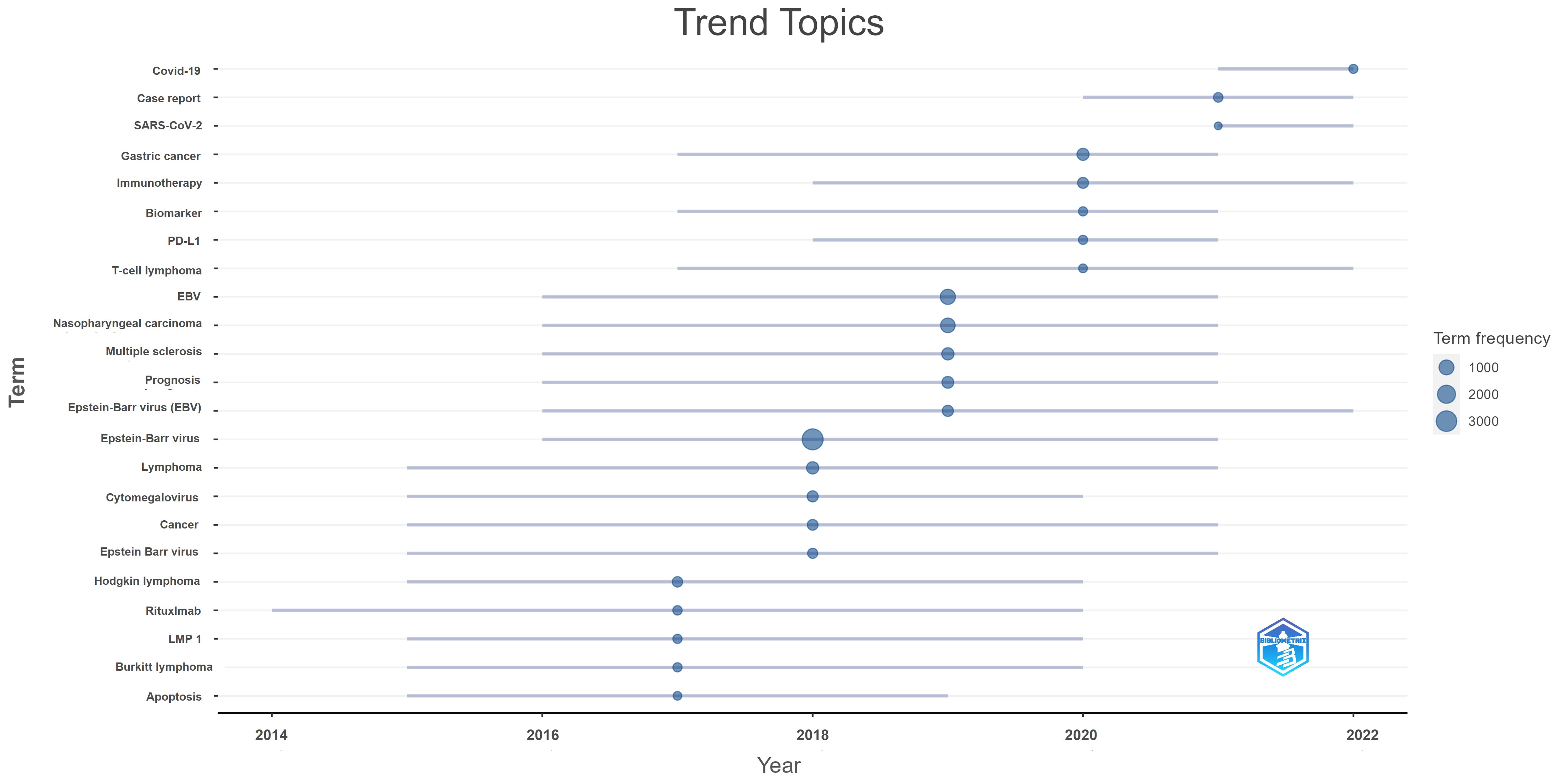
**

**Fig. S6.** Clusters of keywords and timeline of keywords.

**
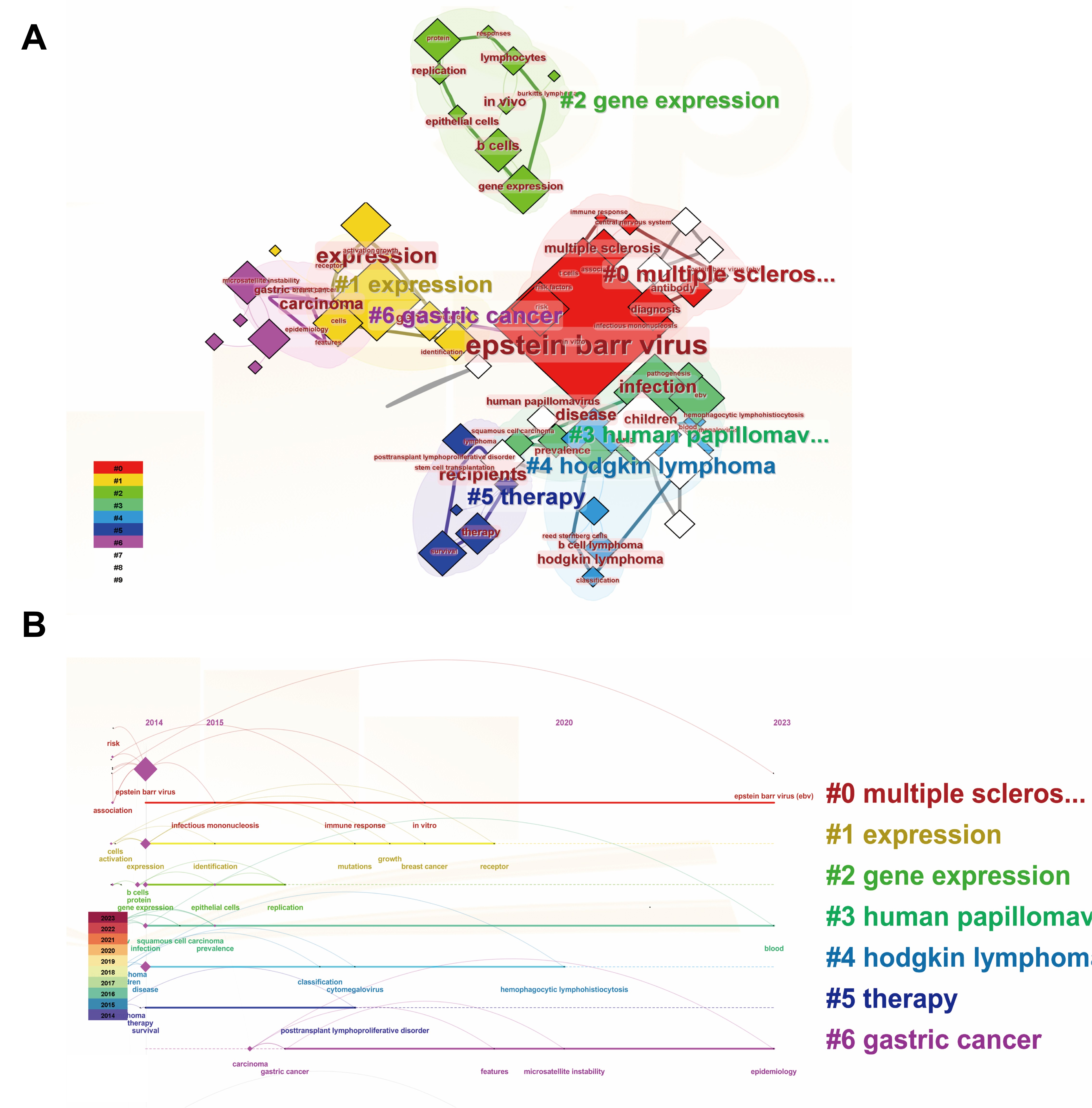
**

**Fig. S7.** The number of research articles and clinical trials about EBV field (1964–2024).

**
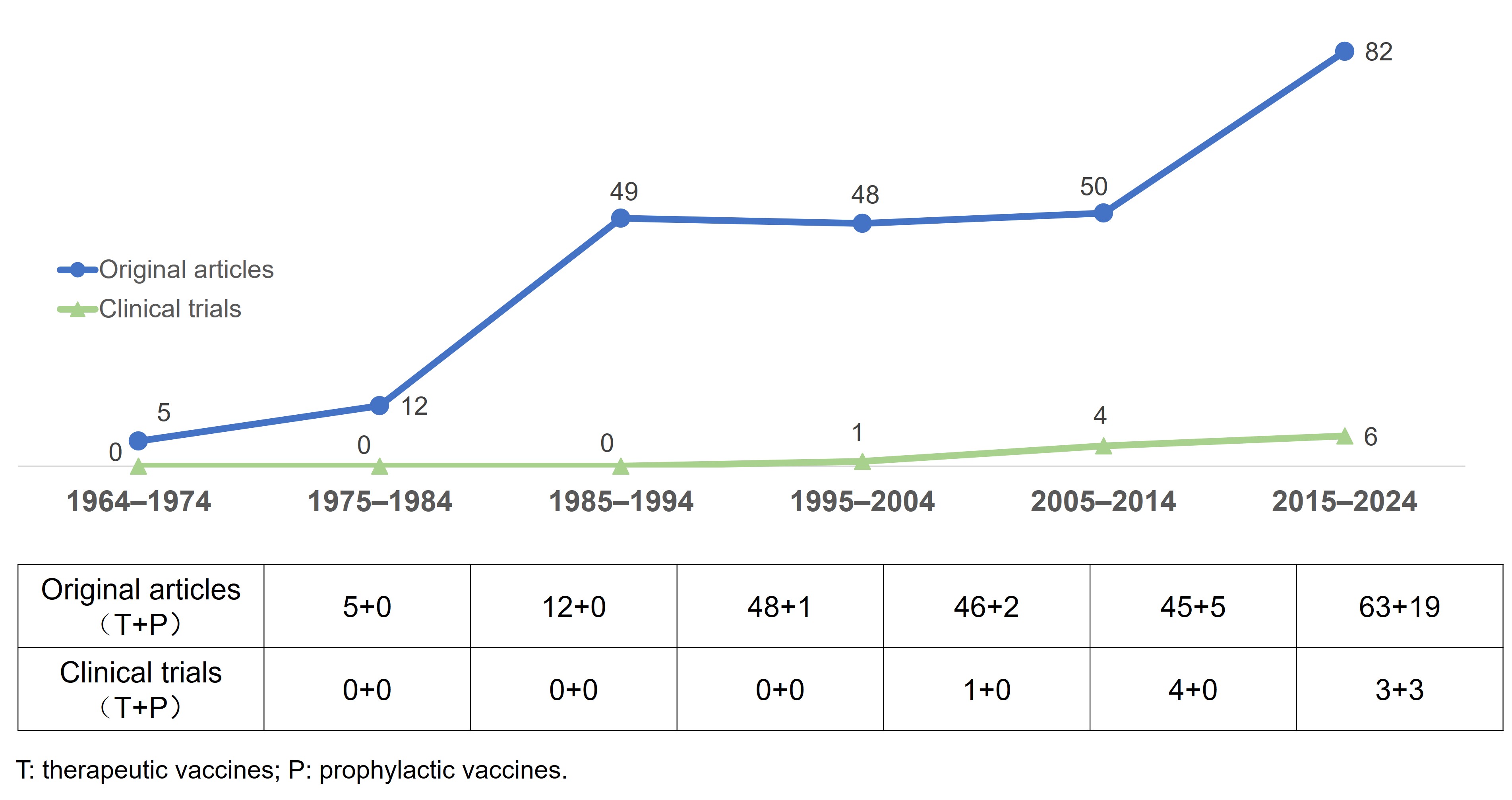
**

**Table S1** The most 10 journals that published EBV researches.

| **Rank** | **Journals** | **Documents, *n* (%)** | **Citations** | **Average citations** | **IF 2023,**  **JCR Quartile** | **Region** |
| --- | --- | --- | --- | --- | --- | --- |
| 1 | Journal of Virology | 398 (2.47%) | 10,051 | 25.25 | 4, Q2 | United States |
| 2 | Frontiers in Immunology | 360 (2.23%) | 7060 | 19.61 | 5.7, Q1 | Switzerland |
| 3 | Plos One | 360 (2.23%) | 8484 | 23.57 | 2.9, Q1 | United States |
| 4 | Cancers | 219 (1.36%) | 2272 | 10.37 | 4.5, Q1 | Switzerland |
| 5 | Plos Pathogens | 206 (1.28%) | 6488 | 31.50 | 5.5, Q1 | United States |
| 6 | Viruses-Basel | 199 (1.23%) | 3207 | 16.12 | 3.8, Q2 | Switzerland |
| 7 | Scientific Reports | 198 (1.23%) | 3198 | 16.15 | 3.8, Q1 | United Kingdom |
| 8 | Frontiers in Oncology | 185 (1.15%) | 2486 | 13.44 | 3.5, Q2 | Switzerland |
| 9 | International Journal of Molecular Sciences | 161 (1.00%) | 1855 | 11.52 | 4.9, Q2 | Switzerland |
| 10 | Medicine | 158 (0.98%) | 1441 | 9.12 | 1.3, Q2 | United States |

IF = Impact Factor, JCR = Journal Citation Reports.

**Table S2** Insights from recent studies.

| Topic | Study year | Key findings | References |
| --- | --- | --- | --- |
| Vaccines | 2023 | The article highlights the development of a gB nanoparticle vaccine against EBV, gB-I53-50 NP, displays multiple copies of the gB protein. This rational design shows enhanced structural stability and immunogenicity. It induces a robust and durable protective antibody response in mice and non-human primates, effectively shielding humanized mice from lethal EBV challenges. | 49 |
|  | 2022 | The article presents a self-assembling nanoparticle vaccine displaying the gH/gL protein complex of EBV. It highlights the ability to induce potent neutralizing antibody responses in humanized mice, providing protection against lethal EBV challenges. | 50 |
|  | 2022 | The article describes the development of a bivalent EBV vaccine, which uses single-chain gH/gL and gH/gL/gp42 proteins fused to ferritin to form self-assembling nanoparticles. These nanoparticles effectively induce neutralizing antibodies in various animal models, including mice, ferrets, and nonhuman primates, blocking EBV entry into target cells. | 51 |
| Neutralizing antibodies | 2022 | The article discusses the development of protective neutralizing antibodies against EBV by targeting two vulnerable sites on the gB protein. The study identifies specific regions on gB that are essential for the fusion process and can be targeted to prevent viral entry. This approach offers a potential strategy for developing vaccines or therapies that can prevent or treat EBV infections. | 52 |
|  | 2023 | Three neutralizing antibodies (nAbs) targeting different glycoproteins of the gHgL-gp42 complex were reported. The combination of these three antibodies had synergistic neutralizing effects against B cell infection, and the combination of two of them showed synergistic effects against epithelial cell infection. It demonstrated strong protective effects of the antibody combinations against lethal EBV challenge in humanized mice. Cryo-EM structural analysis revealed non-overlapping epitopes of these three antibodies on the gHgL-gp42 complex, highlighting their distinct neutralizing mechanisms. | 53 |
|  | 2021 | This article reports a human neutralizing antibody recognizing EBV gH/gL, 1D8, which was isolated monoclonal antibodies from EBV-infected individuals' B lymphocytes. 1D8 showed high efficacy in neutralizing EBV infection of B cells and epithelial cells. In the humanized mouse model, 1D8 provided protection against high-dose EBV challenge by significantly reducing viral load and tumor burden. Crystal structure analysis revealed 1D8 binds to a key vulnerable interface between D-I/D-II domains of gH/gL protein. | 54 |
| Structure of viral proteins and small molecule drug design | 2022 | The study provides high-resolution insights into the EBV capsid, including the flexibility of its pentons, the closed conformation of the portal, unique portal-DNA interactions, and the stoichiometric binding of the capsid-associated tegument complex (CATC). These structural features offer new functional roles for the capsid penton vertices, portal, and CATCs, shedding light on the processes related to viral genome packaging, retention, and ejection. | 55 |
|  | 2024 | The research reveals the structural and functional insights into the latent membrane protein 1 (LMP1) of EBV, a primary oncoprotein implicated in the viral lifecycle and pathogenesis. This study employs cryo-electron microscopy to uncover the unexpected assembly forms of LMP1, including dimers and filamentous oligomers, which are essential for its role in disease. It will be potential therapeutic by disrupting these assemblies of LMP1. | 56 |
|  | 2019 | Using a fragment-based approach and X-ray crystallography, researchers identify a 2,3-disubstituted benzoic acid series that selectively inhibits the DNA binding activity of EBNA1. These inhibitors selectively block EBV gene transcription and alter the cellular transforming growth factor-β (TGF-β) signaling pathway in NPC tumor xenografts. | 57 |
| EBV infection and receptors | 2024 | The article explores the role of IL-27 in EBV infection through IL-27RA deficiency. It finds that IL27RA mutations cause severe primary EBV infection but have a good prognosis. IL-27RA deficiency impairs T-cell proliferation and the expansion of anti-EBV cytotoxic CD8^+^ T cells. The study also identifies neutralizing anti-IL-27 autoantibodies in individuals with severe EBV infections, highlighting the dual role of IL-27 in immunity and viral pathogenesis. | 58 |
|  | 2018 | The article reveals that Ephrin receptor A2 (EphA2) is a key receptor for EBV entry into epithelial cells. Mechanistic studies showed that EphA2 interacts with EBV entry proteins gH/gL and gB, promoting EBV endocytosis and fusion. Further research indicated that the extracellular EBD and FNR domains of EphA2 are crucial for mediating EBV infection, while the intracellular domain is dispensable. | 59 |
| Interaction between EBV and host cells | 2024 | The article investigates the metabolic dependencies of EBV in B cell transformation. It reveals that EBV infection activates the tryptophan-kynurenine metabolic pathway, leading to increased NAD de novo synthesis and upregulation of the enzyme IDO1. IDO1 is identified as a key metabolic checkpoint in EBV-induced B cell transformation. a potential therapeutic target for EBV-related diseases. | 60 |
|  | 2023 | Using dynamic single-cell mapping, researchers found that EBV infection leads to T-cell exhaustion, characterized by high expression of inhibitory receptors and functional impairment. This study provides insights into optimizing treatments for EBV-related disease | 61 |
|  | 2024 | The article focuses on stem cell memory EBV-specific T cells and their role in controlling EBV tumor growth and persisting *in vivo*. The findings offer a new direction for the next generation of potent unmodified antigen-specific cell therapies, potentially treating EBV-related diseases, including tumors and other conditions. | 62 |
| EBV and autoimmune diseases | 2022 | The article presents a longitudinal analysis revealing a high prevalence of EBV associated with multiple sclerosis (MS). The risk of developing MS increased 32-fold following EBV infection. Additionally, serum levels of neurofilament light chain (a biomarker of neuronal axonal damage) only increased after EBV seroconversion. | 63 |
|  | 2023 | The article investigates the cross-reactive immunity between Epstein-Barr virus nuclear antigen 1 (EBNA1) and alpha-crystallin B (CRYAB), and its association with multiple sclerosis (MS). The study provides evidence for antibody cross-reactivity between EBNA1 and CRYAB and points to similar cross-reactivity in T cells, further demonstrating the role of EBV adaptive immune responses in the development of MS. | 64 |
| EBV and gastric carcinoma | 2020 | Genome-wide analysis of 3D chromatin topologies across gastric cancer (GC) lines, primary tissue and normal gastric samples revealed chromatin domains specific to EBV-positive GC, exhibiting heterochromatin-to-euchromatin transitions and long-range human-viral interactions with non-integrated EBV episomes. Higher-order epigenotypes of EBV-positive GC thus signify a novel oncogenic paradigm whereby non-integrative viral genomes can directly alter host epigenetic landscapes, facilitating proto-oncogene activation and tumorigenesis. | 65 |
|  | 2022 | The study investigates how a frameshift mutation in the ASTE1 gene triggers an immune response in Epstein-Barr virus-associated gastric cancer (EBVaGC) and its correlation with prognosis. The findings suggest that the ASTE1 mutation is associated with an immune-active phenotype. Additionally, the mutation activates the NF-κB pathway. These insights offer a new perspective on the tumor immune microenvironment in EBVaGC and potential targets for future immunotherapies. | 66 |

*Abbreviations*: NPC, nasopharyngeal carcinoma; LMP, latent membrane protein; CATC, capsid-associated tegument complex; FNR, fibronectin-type III repeat; NAD, nicotinamide adenine dinucleotide; GC, gastric cancer.

**Table S3** Clinical trials for therapeutic vaccines targeting EBV.

| ID | Institution | Vaccine | Antigen | Research objective | Clinical stage | Study start |
| --- | --- | --- | --- | --- | --- | --- |
| NCT00078494 | National Institutes of Health Clinical Center | Ad5-EBV-LMP2 | EBV-LMP2 | Patients at high risk for recurrence of anaplastic nasopharyngeal cancer | Ⅰ | 2004-02 |
| NCT01147991 | Cancer Research United Kingdom | MVA-EBNA1 C-terminal/LMP2 | EBNA1 C-terminal/LMP2 | Patients with Epstein-Barr virus and cancer | Ⅰ | 2005-03 |
| NCT01256853 | The Chinese University of Hong Kong | MVA-EBNA1/LMP2 | EBNA1/LMP2 | Patients in remission having had an EBV+ nasopharyngeal carcinoma | Ⅰ | 2006-09 |
| NCT01094405 | The Chinese University of Hong Kong | MVA EBNA1/LMP2 | EBNA1/LMP2 | Patients with persistent, recurrent or metastatic nasopharyngeal carcinoma | Ⅱ | 2010-03-31 |
| NCT01800071 | Cancer Research United Kingdom | MVA-EBNA1/LMP2 | EBNA1/LMP2 | Patients with EBV+ nasopharyngeal carcinoma | Ⅰ | 2013-03 |
| NCT02115126 | David Rizzieri | LMP2A-loaded conventional DC vaccine | LMP2A | Patients with EBV+ lymphoma in the setting of autologous stem cell transplant with infusion of mature T cells. | Ⅱ | 2016-12 |
| NCT05714748 | West China Hospital | mRNA vaccine | EBV oncoproteins | Patients with EBV-positive advanced malignant tumors | Ⅰ | 2022-11-18 |
| NCT05831111 | Moderna | mRNA-1195 | − | 18- to 55-year-old healthy adults | Ⅰ | 2023-04-05 |

*Abbreviations*: LMP, latent membrane protein; EBNA, Epstein-Barr nuclear antigen; MVA, modified vaccinia Ankara.

**Table S**4 Clinical trials for prophylactic vaccines against EBV.

| ID | Institution | Vaccine | Antigen | Research objective | Clinical stage | Study start |
| --- | --- | --- | --- | --- | --- | --- |
| NCT05164094 | Moderna | mRNA-1189 | gH, gL, gp42, gp220 | 18- to 30-year-old healthy adults and 12- to < 18-year-old EBV-seronegative healthy adolescents. | Ⅰ | 2021-12-28 |
| NCT04645147 | National Institute of Allergy and Infectious Diseases | EBV gp350-Ferritin Vaccine | gp350 | Healthy adults aged 18 to 29 years. | Ⅰ | 2022-03-29 |
| NCT05683834 | National Institute of Allergy and Infectious Diseases | EBV gp350-Ferritin Vaccine | gp350 | Healthy people aged 18 to 25 years. | Ⅰ/Ⅱ | 2023-09-22 |
